# Supplementary material for: Association between serum retinol and overall and cause-specific mortality in a 30-year prospective cohort study
Source: Nat Commun. 2021 Nov 5;12:6418. doi: 10.1038/s41467-021-26639-4 (PMC8571275; doi:10.1038/s41467-021-26639-4)
Supplement: Supplementary file 1 — Supplementary Information [file 41467_2021_26639_MOESM1_ESM.pdf]

Association Between Serum Retinol and Overall and Cause-Specific Mortality in a 30-Year Prospective Cohort Study

Huang et al.

Supplemental Table 1. Hazard ratios (HRs) and 95% CI for CVD mortality by ~~quintile of~~ serum retinol (per one SD), stratified by selected factors

	**HR (95% CI)** [a]	***P* for interaction** [b]
**Age**		
<54 y	0.98 (0.93, 1.02)	0.25
54-59 y	0.94 (0.91, 0.98)	
≥59 y	0.95 (0.91, 0.98)	
**Cigarettes smoked/d**		
<16	0.97 (0.94, 1.01)	0.44
16-20	0.94 (0.90, 0.97)	
>20	0.96 (0.92, 0.99)	
**Daily ethanol consumption (g)**		
<5.3	0.98 (0.94, 1.02)	0.26
5.3-20.4	0.94 (0.90, 0.97)	
≥20.4	0.94 (0.91, 0.98)	
**BMI (kg/m^2)**		
<25	0.97 (0.93, 1.01)	0.21
25-28	0.93 (0.90, 0.97)	
≥28	0.96 (0.92, 0.99)	
**Trial intervention group**		
Alpha-tocopherol	0.96 (0.93, 0.99)	0.74
No alpha-tocopherol	0.95 (0.92, 0.98)	
Beta-carotene	0.94 (0.91, 0.96)	0.020
No beta-carotene	0.97 (0.94, 1.00)	
**Serum alpha-tocopherol**		
Low	0.94 (0.90, 0.98)	0.16
Medium	0.94 (0.90, 0.98)	
High	0.99 (0.96, 1.03)	
**Serum beta-carotene**		
Low	0.95 (0.92, 0.98)	0.36
Medium	0.95 (0.92, 0.99)	
High	0.93 (0.90, 0.98)	
**Serum total cholesterol**		
Low	0.93 (0.89, 0.97)	0.10
Medium	0.94 (0.90, 0.97)	
High	0.99 (0.95, 1.02)	
**History of CVD at baseline**		
No	0.93 (0.90, 0.96)	0.24
Yes	0.98 (0.95, 1.01)	
**Systolic and diastolic blood pressure**		
<130	0.93 (0.88, 0.98)	0.94
130-<150	0.94 (0.91, 0.97)	
≥150	0.95 (0.92, 0.98)	
**Diastolic blood pressure**		
<80	0.92 (0.87, 0.98)	0.21
80-<90	0.91 (0.88, 0.95)	
≥90	0.96 (0.94, 0.99)	
**Years of follow-up**		
0-13 y	0.94 (0.91, 0.97)	0.83
13-23 y	0.96 (0.93, 0.99)	
≥23 y	0.97 (0.92, 1.02)	

Abbreviations: BMI= body mass index; CI= confidence interval; CVD= cardiovascular disease; HDL= high-density lipoprotein; SD= standard deviation

[a] Adjusted for age, BMI, serum total and serum HDL cholesterol, cigarettes smoked per day, years of smoking, alcohol intake, intervention assignment, systolic and diastolic blood pressure, history of CVD, and history of diabetes.

[b] *P* value for interaction: according to the likelihood test to assess the statistical significance of the cross-product term entered into the Cox proportional hazard regression model.

Supplemental Table 2. Hazard ratios (HRs) and 95% CI for heart disease mortality by ~~quintile of~~ serum retinol (per one SD), stratified by selected factors

	**HR (95% CI)** [a]	***P* for interaction** [b]
**Age**		
<54 y	0.97 (0.92, 1.02)	0.19
54-59 y	0.92 (0.89, 0.96)	
≥59 y	0.95 (0.92, 0.99)	
**Cigarettes smoked/d**		
<16	0.96 (0.92, 1.00)	0.64
16-20	0.94 (0.90, 0.98)	
>20	0.95 (0.91, 0.99)	
**Daily ethanol consumption (g)**		
<5.3	0.96 (0.91, 1.00)	0.79
5.3-20.4	0.93 (0.89, 0.98)	
≥20.4	0.94 (0.90, 0.98)	
**BMI (kg/m^2)**		
<25	0.98 (0.94, 1.02)	0.048
25-28	0.91 (0.87, 0.95)	
≥28	0.96 (0.92, 0.99)	
**Trial intervention group**		
Alpha-tocopherol	0.95 (0.92, 0.99)	0.43
No alpha-tocopherol	0.94 (0.91, 0.97)	
Beta-carotene	0.92 (0.89, 0.95)	0.0083
No beta-carotene	0.97 (0.94, 1.01)	
**Serum alpha-tocopherol**		
Low	0.93 (0.89, 0.98)	0.17
Medium	0.93 (0.89, 0.97)	
High	0.98 (0.95, 1.02)	
**Serum beta-carotene**		
Low	0.94 (0.91, 0.98)	0.58
Medium	0.94 (0.90, 0.98)	
High	0.93 (0.89, 0.98)	
**Serum total cholesterol**		
Low	0.93 (0.89, 0.98)	0.27
Medium	0.92 (0.88, 0.96)	
High	0.97 (0.94, 1.01)	
**History of CVD at baseline**		
No	0.90 (0.87, 0.94)	0.075
Yes	0.98 (0.95, 1.01)	
**Systolic and diastolic blood pressure**		
<130	0.90 (0.85, 0.95)	0.46
130-<150	0.93 (0.90, 0.97)	
≥150	0.96 (0.93, 1.00)	
**Diastolic blood pressure**		
<80	0.92 (0.86, 0.98)	0.09
80-<90	0.90 (0.86, 0.94)	
≥90	0.97 (0.93, 1.00)	
**Years of follow-up**		
0-13 y	0.94 (0.91, 0.98)	0.93
13-23 y	0.95 (0.91, 0.98)	
≥23 y	0.95 (0.90, 1.01)	

Abbreviations: BMI= body mass index; CI= confidence interval; CVD= cardiovascular disease; HDL= high-density lipoprotein; SD= standard deviation

[a] Adjusted for age, BMI, serum total and serum HDL cholesterol, cigarettes smoked per day, years of smoking, alcohol intake, intervention assignment, systolic and diastolic blood pressure, history of CVD, and history of diabetes.

[b] *P* value for interaction: according to the likelihood test to assess the statistical significance of the cross-product term entered into the Cox proportional hazard regression model.

Supplemental Table 3. Hazard ratios (HRs) and 95% CI for respiratory disease mortality by ~~quintile of~~ serum retinol (per one SD), stratified by selected factors

	**HR (95% CI)** [a]	***P* for interaction** [b]
**Age**		
<54 y	0.90 (0.80, 1.01)	
54-59 y	0.84 (0.77, 0.91)	0.19
≥59 y	0.91 (0.85, 0.98)	
**Cigarettes smoked/d**		
<16	0.89 (0.81, 0.98)	
16-20	0.90 (0.83, 0.98)	0.88
>20	0.88 (0.81, 0.95)	
**Daily ethanol consumption (g)**		
<5.3	0.90 (0.82, 1.00)	
5.3-20.4	0.90 (0.82, 0.98)	0.67
≥20.4	0.86 (0.79, 0.93)	
**BMI (kg/m^2)**		
<25	0.92 (0.86, 0.99)	
25-28	0.88 (0.80, 0.97)	0.63
≥28	0.86 (0.78, 0.96)	
**Trial intervention group**		
Alpha-tocopherol	0.87 (0.81, 0.93)	0.81
No alpha-tocopherol	0.89 (0.83, 0.96)	
Beta-carotene	0.93 (0.86, 0.99)	0.064
No beta-carotene	0.84 (0.78, 0.90)	
**Serum alpha-tocopherol**		
Low	0.90 (0.84, 0.97)	
Medium	0.83 (0.76, 0.91)	0.076
High	0.98 (0.89, 1.07)	
**Serum beta-carotene**		
Low	0.83 (0.77, 0.89)	
Medium	0.91 (0.84, 1.00)	0.43
High	0.93 (0.85, 1.02)	
**Serum total cholesterol**		
Low	0.85 (0.79, 0.92)	
Medium	0.87 (0.80, 0.95)	0.18
High	0.97 (0.88, 1.06)	
**History of CVD at baseline**		
No	0.85 (0.80, 0.91)	0.50
Yes	0.92 (0.85, 0.99)	
**Years of follow-up**		
0-13 y	0.82 (0.75, 0.89)	
13-23 y	0.89 (0.84, 0.96)	0.14
≥23 y	0.93 (0.84, 1.02)	

Abbreviations: BMI= body mass index; CI= confidence interval; CVD= cardiovascular disease; HDL= high-density lipoprotein; SD= standard deviation

[a] Adjusted for age, BMI, serum total and serum HDL cholesterol, cigarettes smoked per day, years of smoking, alcohol intake, intervention assignment, systolic and diastolic blood pressure, history of CVD, and history of diabetes.

[b] *P* value for interaction: according to the likelihood test to assess the statistical significance of the cross-product term entered into the Cox proportional hazard regression model.

Supplemental Table 4. Multivariate-adjusted hazard ratios (HRs) for overall and cause-specific mortality by quintile of serum retinol in the ATBC Study, excluding the participants who reported a history of CVD or diabetes mellitus at baseline [a] (We used Cox proportional hazard regression models to estimate HRs and 95% CIs. All reported P-values are 2-sided at type I error rate of 0.05. The Bonferroni correction threshold was applied to control for multiple tests (0.05/9=0.0056 [nine tests])

**Cause of death**	Serum retinol, HR (95% CI)						
	Quintile 1	Quintile 2	Quintile 3	Quintile 4	Quintile 5	*P* value for trend [b]	Per one SD (130 µg/L)
Overall mortality	1.0	0.87 (0.83, 0.92)	0.83 (0.79, 0.88)	0.78 (0.74, 0.83)	0.81 (0.76, 0.86)	<0.0001	0.94 (0.92, 0.95)
CVD	1.0	0.76 (0.69, 0.83)	0.77 (0.71, 0.85)	0.70 (0.64, 0.77)	0.72 (0.66, 0.80)	<0.0001	0.92 (0.89, 0.96)
Heart disease	1.0	0.73 (0.66, 0.81)	0.75 (0.68, 0.83)	0.68 (0.61, 0.75)	0.68 (0.61, 0.76)	<0.0001	0.90 (0.87, 0.93)
Stroke	1.0	0.87 (0.71, 1.08)	0.88 (0.71, 1.09)	0.83 (0.66, 1.03)	0.94 (0.75, 1.17)	0.57	1.01 (0.94, 1.09)
Cancer	1.0	0.99 (0.90, 1.08)	0.94 (0.86, 1.03)	0.91 (0.83, 1.00)	0.92 (0.83, 1.02)	0.037	0.97 (0.94, 1.00)
Respiratory disease	1.0	0.82 (0.70, 0.96)	0.77 (0.65, 0.90)	0.71 (0.59, 0.84)	0.60 (0.49, 0.73)	<0.0001	0.85 (0.80, 0.91)
Diabetes mellitus	1.0	0.33 (0.088, 1.26)	0.45 (0.13, 1.50)	0.53 (0.17, 1.68)	0.86 (0.28, 2.59)	0.97	0.98 (0.64, 1.51)
Injuries and accidents	1.0	0.94 (0.75, 1.18)	0.72 (0.56, 0.92)	0.88 (0.69, 1.11)	0.92 (0.72, 1.18)	0.52	0.99 (0.91, 1.07)
Other causes	1.0	0.96 (0.82, 1.11)	0.85 (0.73, 1.00)	0.74 (0.63, 0.87)	0.90 (0.77, 1.06)	0.045	0.93 (0.88, 0.99)

Abbreviations: BMI= body mass index; CI= confidence interval; CVD= cardiovascular disease; HDL= high-density lipoprotein; SD= standard deviation

[a] Adjusted for age, BMI, serum total and serum HDL cholesterol, cigarettes smoked per day, years of smoking, alcohol intake, intervention assignment, systolic and diastolic blood pressure. This analysis included 16,616 subjects after excluding participants who reported a history of CVD or diabetes mellitus at baseline.

[b] *P* value for trend: based on statistical significance of the coefficient of the quintile variable (median value within each quintile).

Supplemental Table 5. Multivariate-adjusted hazard ratios (HRs) for overall and cause-specific mortality by quintile of serum retinol in the ATBC Study, excluding the first 5 years of follow-up [a] (We used Cox proportional hazard regression models to estimate HRs and 95% CIs. All reported P-values are 2-sided at type I error rate of 0.05. The Bonferroni correction threshold was applied to control for multiple tests (0.05/9=0.0056 [nine tests])

**Cause of death**	Serum retinol, HR (95% CI)						
	Quintile 1	Quintile 2	Quintile 3	Quintile 4	Quintile 5	*P* value for trend [b]	Per one SD (130 µg/L)
Overall mortality	1.00	0.88 (0.84, 0.91)	0.84 (0.81, 0.88)	0.81 (0.78, 0.85)	0.84 (0.81, 0.88)	<0.0001	0.95 (0.94, 0.97)
CVD	1.00	0.82 (0.77, 0.88)	0.84 (0.79, 0.90)	0.76 (0.71, 0.82)	0.81 (0.75, 0.86)	<0.0001	0.95 (0.93, 0.98)
Heart disease	1.00	0.82 (0.76, 0.88)	0.84 (0.78, 0.91)	0.75 (0.70, 0.82)	0.79 (0.73, 0.85)	<0.0001	0.95 (0.92, 0.97)
Stroke	1.00	0.85 (0.73, 0.99)	0.85 (0.73, 1.00)	0.81 (0.69, 0.95)	0.89 (0.76, 1.04)	0.21	0.98 (0.93, 1.04)
Cancer	1.00	0.96 (0.89, 1.04)	0.91 (0.84, 0.98)	0.91 (0.84, 0.98)	0.94 (0.87, 1.02)	0.093	0.98 (0.95, 1.00)
Respiratory disease	1.00	0.83 (0.73, 0.94)	0.74 (0.64, 0.84)	0.76 (0.66, 0.87)	0.68 (0.58, 0.79)	<0.0001	0.89 (0.84, 0.93)
Diabetes mellitus	1.00	0.69 (0.36, 1.30)	0.85 (0.47, 1.57)	0.99 (0.56, 1.76)	0.96 (0.53, 1.72)	0.71	1.06 (0.87, 1.27)
Injuries and accidents	1.00	0.97 (0.79, 1.19)	0.79 (0.64, 0.98)	0.92 (0.75, 1.13)	0.99 (0.80, 1.21)	0.94	1.01 (0.95, 1.08)
Other causes	1.00	0.89 (0.79, 1.01)	0.82 (0.73, 0.93)	0.76 (0.67, 0.87)	0.85 (0.74, 0.96)	0.0045	0.93 (0.89, 0.97)

Abbreviations: BMI= body mass index; CI= confidence interval; CVD= cardiovascular disease; HDL= high-density lipoprotein; SD= standard deviation

[a] Adjusted for age, BMI, serum total and serum HDL cholesterol, cigarettes smoked per day, years of smoking, alcohol intake, intervention assignment, systolic and diastolic blood pressure, history of CVD, and history of diabetes. This analysis included 26,377subjects after excluding the first 5 years of follow-up.

[b] *P* value for trend: based on statistical significance of the coefficient of the quintile variable (median value within each quintile).

Supplemental Table 6. Multivariate-adjusted hazard ratios (HRs) for overall and cause-specific mortality by quintile of serum retinol concentration at three years in the ATBC Study [a] (We used Cox proportional hazard regression models to estimate HRs and 95% CIs. All reported P-values are 2-sided at type I error rate of 0.05. Bonferroni corrected P_{trend} value is also presented)

Causes of mortality	Serum retinol (mg/L)						
	Quintile 1	Quintile 2	Quintile 3	Quintile 4	Quintile 5	P for trend	Bonferroni corrected P for trend
**All-cause**							
Deaths (n)	3180	3415	3301	3578	4196		
Age-adjusted HR (95% CI) [b]	1.00	0.89 (0.84, 0.93)	0.85 (0.81, 0.89)	0.84 (0.80, 0.88)	0.90 (0.86, 0.94)	0.0002	0.0018
Multivariate HR (95% CI) [c]	1.00	0.88 (0.84, 0.93)	0.84 (0.80, 0.89)	0.82 (0.78, 0.86)	0.85 (0.81, 0.89)	<0.0001	<0.0001
**CVD**							
Deaths (n)	1209	1377	1359	1473	1769		
Age-adjusted HR (95% CI) [b]	1.00	0.95 (0.88, 1.02)	0.93 (0.86, 1.01)	0.92 (0.86, 1.00)	1.01 (0.94, 1.09)	0.47	1.00
Multivariate HR (95% CI) [c]	1.00	0.91 (0.85, 0.99)	0.89 (0.82, 0.96)	0.85 (0.79, 0.92)	0.88 (0.82, 0.95)	0.0016	0.01
**Heart disease**							
Deaths (n)	1000	1114	1112	1202	1421		
Age-adjusted HR (95% CI) [b]	1.00	0.93 (0.85, 1.01)	0.92 (0.85, 1.01)	0.91 (0.84, 0.99)	0.98 (0.91, 1.07)	0.99	1.00
Multivariate HR (95% CI) [c]	1.00	0.89 (0.82, 0.97)	0.87 (0.80, 0.95)	0.83 (0.76, 0.90)	0.84 (0.77, 0.91)	0.0001	0.0009
**Stroke**							
Deaths (n)	200	258	244	264	338		
Age-adjusted HR (95% CI) [b]	1.00	1.07 (0.89, 1.29)	1.01 (0.84, 1.22)	1.00 (0.83, 1.21)	1.18 (0.99, 1.40)	0.091	0.82
Multivariate HR (95% CI) [c]	1.00	1.07 (0.89, 1.29)	1.02 (0.84, 1.23)	0.99 (0.82, 1.19)	1.12 (0.93, 1.34)	0.35	1.00
**Cancer**							
Deaths (n)	1029	1141	1088	1201	1371		
Age-adjusted HR (95% CI) [b]	1.00	0.91 (0.84, 0.99)	0.87 (0.80, 0.94)	0.87 (0.80, 0.95)	0.90 (0.83, 0.98)	0.024	0.22
Multivariate HR (95% CI) [c]	1.00	0.92 (0.84, 1.00)	0.88 (0.80, 0.95)	0.87 (0.80, 0.95)	0.90 (0.82, 0.97)	0.019	0.17
**Respiratory disease**							
Deaths (n)	391	342	316	309	307		
Age-adjusted HR (95% CI) [b]	1.00	0.73 (0.63, 0.84)	0.68 (0.58, 0.78)	0.61 (0.52, 0.71)	0.56 (0.48, 0.65)	<0.0001	<0.0001
Multivariate HR (95% CI) [c]	1.00	0.77 (0.67, 0.90)	0.74 (0.64, 0.86)	0.67 (0.57, 0.78)	0.62 (0.53, 0.72)	<0.0001	<0.0001
**Diabetes mellitus**							

Deaths (n)	16	17	16	19	28		
Age-adjusted HR (95% CI) [b]	1.00	0.87 (0.44, 1.73)	0.82 (0.41, 1.64)	0.89 (0.46, 1.74)	1.20 (0.65, 2.23)	0.73	1.00
Multivariate HR (95% CI) [c]	1.00	0.89 (0.45, 1.77)	0.72 (0.36, 1.45)	0.80 (0.41, 1.56)	0.96 (0.51, 1.82)	0.97	1.00
**Injuries and accidents**							
Deaths (n)	129	166	130	164	236		
Age-adjusted HR (95% CI) [b]	1.00	1.05 (0.83, 1.32)	0.81 (0.63, 1.03)	0.92 (0.73, 1.16)	1.18 (0.95, 1.46)	0.10	0.90
Multivariate HR (95% CI) [c]	1.00	1.08 (0.85, 1.35)	0.82 (0.64, 1.05)	0.92 (0.73, 1.16)	1.12 (0.89, 1.40)	0.38	1.00
**Other causes**							
Deaths (n)	406	372	392	412	485		
Age-adjusted HR (95% CI) [b]	1.00	0.73 (0.64, 0.85)	0.76 (0.66, 0.87)	0.73 (0.64, 0.84)	0.78 (0.68, 0.89)	0.007	0.06
Multivariate HR (95% CI) [c]	1.00	0.73 (0.64, 0.85)	0.76 (0.66, 0.87)	0.72 (0.62, 0.82)	0.74 (0.64, 0.84)	0.0004	0.004

Abbreviations: ATBC=Alpha-Tocopherol, Beta-Carotene Cancer Prevention; BMI= body mass index; CI= confidence interval; CVD= cardiovascular disease; HDL= high-density lipoprotein

[a] There were 22,312 men included in this sensitivity analysis.

[b] Adjusted for age. *P* value for trend: based on statistical significance of the coefficient of the quintile variable (median value within each quintile).

[c] Adjusted for age, BMI, serum total and serum HDL cholesterol, cigarettes smoked per day, years of smoking, alcohol intake, intervention assignment, systolic and diastolic blood pressure, history of CVD, and history of diabetes.

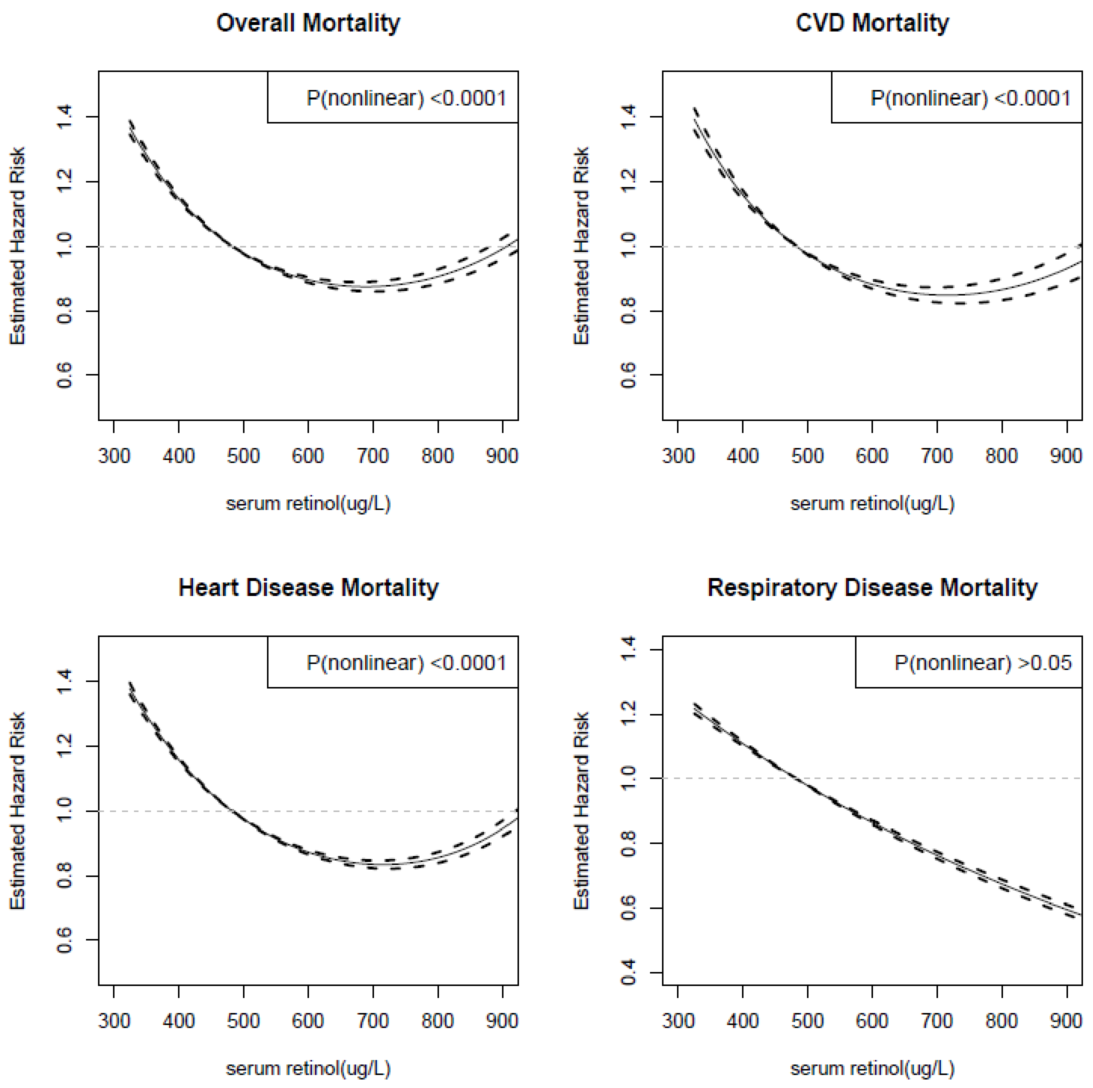

Supplemental Figure 1. Multivariable fractional polynomial models for the nonlinear association between serum retinol and overall and cause-specific death in the ATBC Study. The reference value (483 µg/L; hazard ratio = 1) corresponds to the cutoff value of the first quintile category of serum retinol concentration. A) Overall mortality. B) Cardiovascular disease (CVD) mortality. C) Heart disease mortality. D) Respiratory disease mortality. We used multivariable fractional polynomial models adjusted for age, BMI, serum total and serum HDL cholesterol, cigarettes smoked per day, years of smoking, alcohol intake, intervention assignment, systolic and diastolic blood pressure, history of CVD, and history of diabetes. All reported P-values are 2-sided at type I error rate of 0.05.

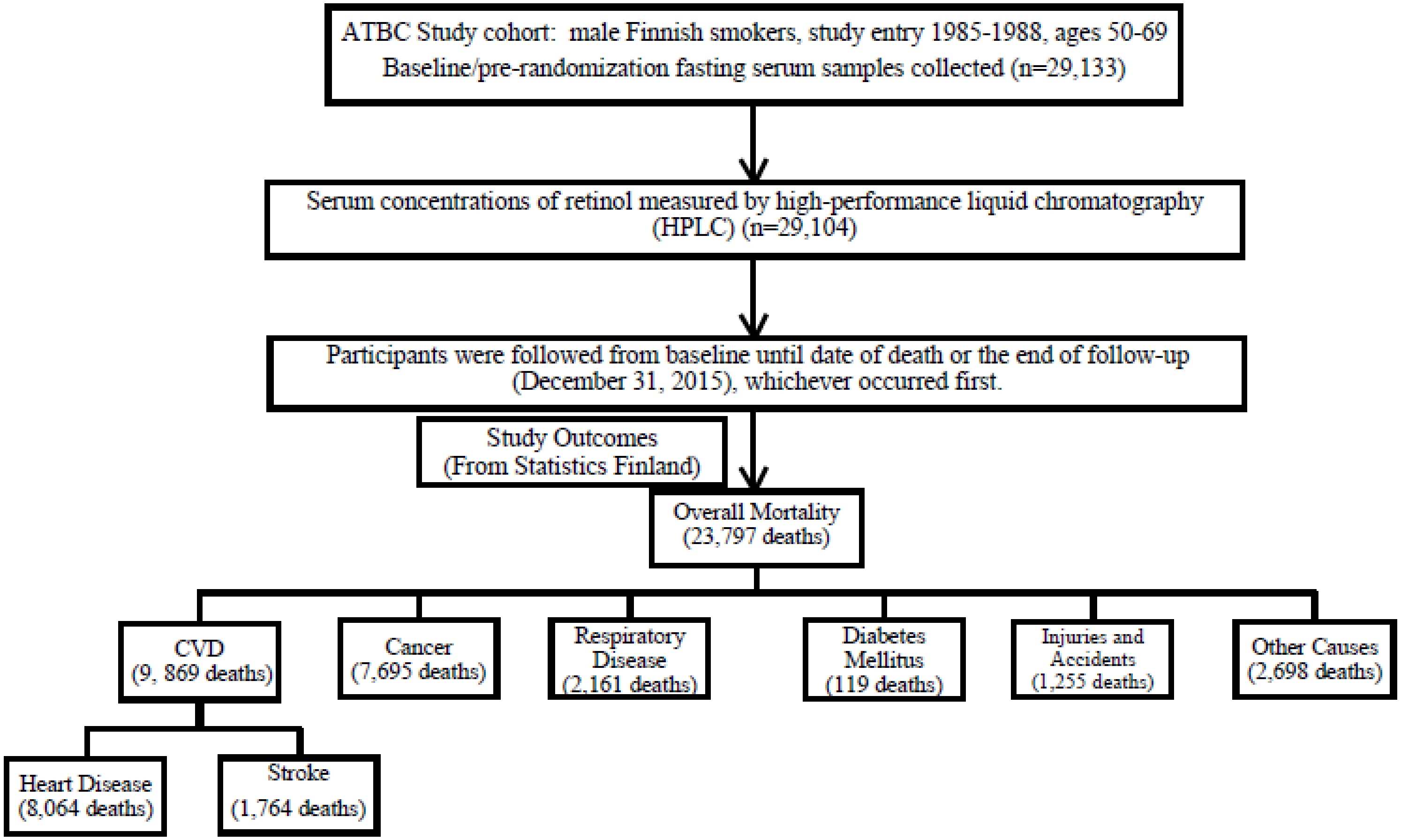

Supplemental Figure 2. Flow chart for study of association between serum retinol and overall and cause-specific mortality in a 30-year prospective analysis in the Alpha-Tocopherol, Beta-Carotene Cancer Prevention (ATBC) Study
