# Supplementary material for: Association between serum retinol and overall and cause-specific mortality in a 30-year prospective cohort study
Source: Nat Commun. 2021 Nov 5;12:6418. doi: 10.1038/s41467-021-26639-4 (PMC8571275; doi:10.1038/s41467-021-26639-4)
Supplement: Supplementary file 3 — Reporting Summary [file 41467_2021_26639_MOESM3_ESM.pdf]

nature portfolio

Corresponding author(s): Jiaqi Huang and Demetrius Albanes

Last updated by author(s): Sep 1, 2021

Reporting Summary

Nature Portfolio wishes to improve the reproducibility of the work that we publish. This form provides structure for consistency and transparency in reporting. For further information on Nature Portfolio policies, see our Editorial Policies and the Editorial Policy Checklist.

Statistics

For all statistical analyses, confirm that the following items are present in the figure legend, table legend, main text, or Methods section.

n/a	Confirmed	
☐	☒	The exact sample size (n) for each experimental group/condition, given as a discrete number and unit of measurement
☐	☒	A statement on whether measurements were taken from distinct samples or whether the same sample was measured repeatedly
☐	☒	The statistical test(s) used AND whether they are one- or two-sided<br>*Only common tests should be described solely by name; describe more complex techniques in the Methods section.*
☐	☒	A description of all covariates tested
☐	☒	A description of any assumptions or corrections, such as tests of normality and adjustment for multiple comparisons
☐	☒	A full description of the statistical parameters including central tendency (e.g. means) or other basic estimates (e.g. regression coefficient) AND variation (e.g. standard deviation) or associated estimates of uncertainty (e.g. confidence intervals)
☐	☒	For null hypothesis testing, the test statistic (e.g. F, t, r) with confidence intervals, effect sizes, degrees of freedom and P value noted<br>*Give P values as exact values whenever suitable.*
☒	☐	For Bayesian analysis, information on the choice of priors and Markov chain Monte Carlo settings
☒	☐	For hierarchical and complex designs, identification of the appropriate level for tests and full reporting of outcomes
☐	☒	Estimates of effect sizes (e.g. Cohen's d, Pearson's r), indicating how they were calculated

Our web collection on statistics for biologists contains articles on many of the points above.

Software and code

Policy information about availability of computer code

Data collection	SAS software (version 9.4; SAS Institute Inc, Cary, NC) and the R statistical language (version 3.5.2; Vienna, Austria).
Data analysis	SAS software (version 9.4; SAS Institute Inc, Cary, NC) and the R statistical language (version 3.5.2; Vienna, Austria)

For manuscripts utilizing custom algorithms or software that are central to the research but not yet described in published literature, software must be made available to editors and reviewers. We strongly encourage code deposition in a community repository (e.g. GitHub). See the Nature Portfolio guidelines for submitting code & software for further information.

Data

Policy information about availability of data

All manuscripts must include a data availability statement. This statement should provide the following information, where applicable:

- Accession codes, unique identifiers, or web links for publicly available datasets
- A description of any restrictions on data availability
- For clinical datasets or third party data, please ensure that the statement adheres to our policy

Because of previously enacted EU Data Protection Regulation (GDPR) privacy rules and an existing data use agreement between Finland and the U.S. National Cancer Institute, the ATBC Study data and materials described in the manuscript may not be made indiscriminately publicly available for the purposes of reproducing the findings. Please contact the ATBC Study Principal Investigators for appropriate specific data requests (https://atbcstudy.cancer.gov/). Data requests are subject to approval by the study review committees. For example, the review committee will check if the data requests containing information that could compromise research participant privacy or participant informed consent. The response time to data requests may be affected during the COVID-19 pandemic.

Field-specific reporting

Please select the one below that is the best fit for your research. If you are not sure, read the appropriate sections before making your selection.

- [x] Life sciences
- [ ] Behavioural & social sciences
- [ ] Ecological, evolutionary & environmental sciences

For a reference copy of the document with all sections, see nature.com/documents/nr-reporting-summary-flat.pdf

Life sciences study design

All studies must disclose on these points even when the disclosure is negative.

Sample size	The sample size of this study was based on number of samples that had retinol measurement data using reversed-phase high-performance liquid chromatography that passed quality control.
Data exclusions	Serum concentrations of retinol, alpha-tocopherol, and beta-carotene were measured for 29,104 of the 29,133 cohort participants (99.9%) using reversed-phase high-performance liquid chromatography. Participants without retinol, alpha-tocopherol, and beta-carotene measurement were excluded from this analysis (0.1%).
Replication	We conducted sensitivity analysis to examine serum retinol concentrations measured from blood collected in year three in relation to subsequent risk of overall and cause-specific mortality to confirm our main findings as a replication analysis.
Randomization	This study is not a randomized trial analysis but rather a prospective cohort analysis.
Blinding	This is an observational study, so blinding is not applicable.

Reporting for specific materials, systems and methods

We require information from authors about some types of materials, experimental systems and methods used in many studies. Here, indicate whether each material, system or method listed is relevant to your study. If you are not sure if a list item applies to your research, read the appropriate section before selecting a response.

Materials & experimental systems

n/a	Involved in the study	
x		Antibodies
x		Eukaryotic cell lines
x		Palaeontology and archaeology
x		Animals and other organisms
	x	Human research participants
x		Clinical data
x		Dual use research of concern

Methods

n/a	Involved in the study	
x		ChIP-seq
x		Flow cytometry
x		MRI-based neuroimaging

Human research participants

Policy information about studies involving human research participants

Population characteristics	There were 29,104 men included (aged 50-69 years), and 23,797 deaths were identified through Finnish registry linkage, including death from cardiovascular disease (9,869; 8,064 from heart disease and 1,764 from stroke), cancer (7,695), respiratory disease (2,161), diabetes (119), injuries and accidents (1,255), and other causes (2,698). Median follow-up time was 18 years. Details regarding the population characteristics for the cohort in this manuscript are provided in Table 1 and Supplemental Figure 2.
Recruitment	Details on the recruitment procedures for the cohort included in this study are provided in the Supplementary Information. Briefly, the study enrolled 29,133 male smokers, aged 50-69 years from 1985 to 1988 in southwestern Finland. To our knowledge, this is the largest analysis to examine the associations between serum retinol (vitamin A) and the risk of overall and cause-specific mortality. This large sample size enabled us to examine associations for the less common causes of mortality and to test whether other factors such as age, alcohol consumption, body mass index, or smoking intensity modified the vitamin A-mortality associations. For the potential biases, we have discussed this in the Discussion section; for example, "The cohort was a relatively homogenous population of male Finnish smokers which limits generalizability of our findings to other populations."
Ethics oversight	The ATBC Study was conducted in accordance with the requirement by the Declaration of Helsinki. The study was reviewed and approved by the Institutional Review Boards at the US National Cancer Institute and the Finnish National Public Health Institute. All participants provided written informed consent.

Note that full information on the approval of the study protocol must also be provided in the manuscript.
